# Pre-validation of a MALDI MS proteomics-based method for the reliable detection of blood and blood provenance

**DOI:** 10.1038/s41598-020-74253-z

**Published:** 2020-10-13

**Authors:** Katie Kennedy, Cameron Heaton, Glenn Langenburg, Laura Cole, Tom Clark, Malcolm R. Clench, Vaughn Sears, Mark Sealey, Richard McColm, Simona Francese

**Affiliations:** 1 Centre for Mass Spectrometry Imaging, Biomolecular Sciences Research Centre, Sheffield Hallam University, Sheffield, UK; 2 Elite Forensic Services, LLC, Saint Paul, MN USA; 3 Sheffield Hallam University, Sheffield, UK; 4 Former Centre for Applied Science and Technology (CAST), Home Office, St Albans, UK; 5 Defence Science and Technology Laboratories (DSTL), Porton Down, Salisbury, UK

**Keywords:** Biological techniques, Biomarkers

## Abstract

The reliable identification of blood, as well as the determination of its origin (human or animal) is of great importance in a forensic investigation. Whilst presumptive tests are rapid and deployed in situ, their very nature requires confirmatory tests to be performed remotely. However, only serological tests can determine blood provenance. The present study improves on a previously devised Matrix Assisted Laser Desorption Ionisation Mass Spectrometry (MALDI MS)—proteomics based method for the reliable detection of blood by enabling the determination of blood provenance. The overall protocol was developed to be more specific than presumptive tests and faster/easier than the gold standard liquid chromatography (LC) MS/MS analysis. This is considered a pre-validation study that has investigated stains and fingermarks made in blood, other biofluids and substances that can elicit a false-positive response to colorimetric or presumptive tests, in a blind fashion. Stains and marks were either untreated or enhanced with a range of presumptive tests. Human and animal blood were correctly discriminated from other biofluids and non-biofluid related matrices; animal species determination was also possible within the system investigated. The procedure is compatible with the prior application of presumptive tests. The refined strategy resulting from iterative improvements through a trial and error study of 56 samples was applied to a final set of 13 blind samples. This final study yielded 12/13 correct identifications with the 13th sample being correctly identified as animal blood but with no species attribution. This body of work will contribute towards the validation of MALDI MS based methods and deployment in violent crimes involving bloodshed.

## Introduction

The reliable detection of bloodstains at the scene of violent crimes is of crucial importance to both reconstruct the dynamics of the crime and to provide, if present, associative evidence. Particularly the presence of blood in fingermarks yields such associative evidence.

Crime scene investigators and forensic laboratories employ a range of blood enhancement techniques (BET) applicable to either stains or fingermarks. As previously reported^1,2^, these methods may be prone to false positives (and are therefore considered presumptive). This is due to the relatively unspecific nature of their molecular targets.

The most commonly known false positive example is the reaction of luminol (haem reactive test) to bleach. A less reported example is represented by the false positive reaction of acid dyes to biofluids other than blood, such as semen and saliva. In this respect, the lack of specificity in the identification of blood is worsened by the risk of not detecting other types of biofluids which are important indicators of the crime being committed. The detection of other biofluids would permit a comprehensive reconstruction of the events. For example, in a homicide crime scene, the presence of semen on the victim, or around a body may indicate that some level of sexual activity has also occurred (from consensual intercourse to rape).

Other important aspects to consider are sequential workflows to gather a body of intelligence from a single evidential source. Blood tests such as the presumptive haem specific Luminol, BlueStar and confirmatory tests such as the Takayama Test, have been shown to result in DNA degradation at 30 days post application; this may be an issue for cases when further DNA analysis is required^3^ but the analysis is not conducted immediately after collection. DNA provides a profile of the individual to whom the blood belongs to (if blood is present). It can relay extremely valuable information to both an investigation and judicial debates but does not provide information on the nature of the biofluid that it may be found within. As such, even if the identity of the perpetrator can be retrieved, the detection of blood may be missed and thus, valuable information may still be lacking around the nature and dynamics of the crime. Due to the limitations of the presumptive tests and DNA techniques, it becomes very important to have validated techniques characterised by high specificity in the detection of blood (and better still of other biofluids also) that, ideally, do not interfere with the subsequent process of DNA sampling and analysis.

Deininger et al. reviewed spectroscopic techniques for blood detection including Raman and hyperspectral imaging (HSI)^2^. Despite these techniques showing great promise for blood detection and distribution mapping, they are not be suitable when blood is recovered from red and dark substrates. Furthermore the need to acquire a reference spectrum at a crime scene without any blood would be challenging, as blood contaminated stains or fingermarks can often be latent at crime scenes.

Mass spectrometry (in different forms) has the advantage of being much more specific compared to spectroscopic methods as it detects the compound of interest by measuring the specific mass-to-charge (*m/z*) ratio; the compound identity can also be confirmed by tandem mass spectrometry experiments. The gold standard for detecting protein biomarkers such as those specific to blood is still currently mass spectrometry-based proteomics through liquid chromatography (LC) hyphenated with Tandem mass spectrometry (LC MS/MS). This is due to both the superior number of protein species detected and the confirmatory value of MS/MS experiments (which can be conducted on single and multiply charged ions). Iliano et al.^4^ reported on the use of LC MS/MS to discriminate different biofluids, by identifying several semen, saliva and blood specific markers. In particular, blood specific proteins detected in the samples analysed included haemoglobin (HB), hemopexin and haptoglobin.

However, LC MS/MS remains rather time consuming from both a sample preparation point of view and data acquisition. In biomarker discovery studies, the benefits outweigh the downsides, due to the necessity to map the entire proteome and much more relaxed time constraints.

Nonetheless, one should carefully evaluate the analytical context and the type of information sought for the selection of the analytical technique to be used. In the context of forensic identification of blood and biofluids, a technique that yields only a handful of blood/biofluid biomarkers, but that is faster and more user friendly, may potentially be more suitable than LC MS/MS.

MALDI MS could represent one such alternative technique. This hypothesis is supported by the work of Yang et al.^5^ in 2013 who devised a MALDI MS and MS/MS method for the differentiation of blood and other bodily fluids. However the method also included prior chromatographic separation, making the whole procedure possibly even more laborious. Jiang et al. uniquely used MALDI FT ICR MS followed by multivariate statistical analysis to differentiate blood and other biofluids^6^. In the study by Kamanna et al.^7^ published in 2017, MALDI MS was employed to detect blood and vaginal fluid biomarkers with or without enhancement using a BET. Blood was confirmed by the presence of haem and HB most abundant peptides (as well as intact HB analysis). Bradshaw et al.^8^ demonstrated in 2014 that MALDI MS Imaging (MALDI MSI) was successful in detecting intact haem and HB visualising them in blood marks with and without prior enhancement. Patel et al. and Deininger et al.^2,9^ further investigated blood detection in human blood stains and blood fingermarks using a MALDI MS and MALDI MSI based proteomic approach. Within both of these studies, the most abundant and specific blood proteins such as haemoglobin α (αHB) and β (βHB), hemopexin, serotransferrin, complement C3, alpha-1 antitrypsin, apolipoprotein A1, alpha-2-macroglobulin, erythrocyte membrane protein band (EPB) 3 and 4.2 were detected, with many of these proteins being highly specific to blood and with EPB 3 being specifically found in the human red blood cell membranes.

The inclusion of multiple blood-specific proteins is a more specific approach compared to those previously described as, although HB is specific to blood, it may be found as a trace contaminant in other biofluids. Furthermore, Bradshaw et al.^8^ Patel et al.^9^ and Kamanna et al.^10^ demonstrated the possibility to determine blood provenance which can be valuable in an investigation.

Taken together these studies indicate MALDI MS Profiling (MALDI MSP) and MALDI MS Imaging (MALDI MSI) based proteomic approaches as a viable and quicker alternative to LC MS/MS methods and as confirmatory tests for both blood detection and provenance. However, despite the potential that MALDI MS has shown for its operational use in blood detection and provenance, no method validation has been conducted, hence the operational implementation both for live and cold cases is somewhat hindered.

In the present study, the authors report on a “pre-validation” study where the methods developed by Patel et al. and Deininger et al.^2,9^ (in which the reduction and alkylation step were removed) are improved and applied to a variety of stains and fingermarks. These samples were deposited on aluminium slides and included blood, other biofluids (human semen, saliva and sweat) and non-biofluids related matrices (ketchup, egg, yolk, body lotion, steak sauce, beetroot juice). All samples were prepared unenhanced or enhanced using three BET namely Acid Yellow-7 (AY-7), Leucocrystal Violet (LCV) and Acid Black 1 (AB-1).

With respect to blood samples, these were either human or animal (porcine (both domesticated and wild boar), bovine and chicken) and in the case of human samples, some contained Ethylenediaminetetraacetic Acid (EDTA) as an example of anti-coagulant. Crucially, all of the samples were prepared blind to the analysts in order to validate the overall method whilst eliminating any possible interpretative bias. The identity of the individual samples was only disclosed once the data were processed and the analyst made the “identity claim”.

An iterative process has been used to “learn” the most efficient way to process the data for the subsequent determination of the nature of the sample “at glance” in a final set of 13 pre-validation samples. Whilst MS/MS has been undertaken to facilitate strategy development, the 13 final pre-validation samples were correctly identified for the presence of human and animal blood, as well as for the presence of semen, without performing MS/MS experiments, thus enabling a quick screening method. This pre-validation work has advised and informed on the best approach for further refinement and optimisation of the study design and methods so that full validation of this MALDI-based approach will be feasible in the near future.

## Materials and methods

All experimental protocols were approved by Sheffield Hallam University (HWB-BRERG23-13-14 and ER13034924) and performed in accordance with relevant guidelines and regulations. An informed consent was obtained from the donor of the human samples prior to sample donation.

### Materials

Trifluoroacetic acid (TFA), α-cyano-4-hydroxycinnamic acid (α-CHCA) and Millipore ZipTips containing C18 stationary phase and TLC sheets were purchased from Sigma Aldrich (Poole, UK). Acetonitrile (ACN) and formic acid were purchased from Fisher Scientific (Loughborough, UK). Sequencing grade modified lyophilized Trypsin was obtained from Promega in 20 µg vials (Southampton, UK). Sigma dry tubed swabs were sourced from Medical Wire (MWE) (Wiltshire, UK) and RapiGest was obtained in 1 mg vials from Waters (Wilmslow, UK). Intravenous blood samples (bovine, chicken and porcine, 1 mL defibrinated and 1 mL with EDTA for each animal) were purchased from TCS Biosciences (Buckingham, UK). All blind stain and fingermark samples were donated by Elite Forensic Services (Minnesota, USA) and used under the Ethics Applications (HWB-BRERG23-13-14 and ER13034924) granted by Sheffield Hallam University. Samples included human and animal blood (bovine, porcine, chicken, and wild boar) human biofluids (semen, saliva and sweat), non-blood/biofluid related matrices (beetroot juice, ketchup, egg white, steak sauce). Human blood, biofluids and fingermarks were donated by 1 male donor at one collection time. Samples were presented both as non-enhanced and enhanced using blood enhancement techniques such as Acid Yellow 7, Acid Black 1 and Leucocrystal Violet and were prepared on TLC aluminium slides. They were subsequently packaged in glass slide containers, kept at room temperature prior to shipping via priority mail to the research group at Sheffield Hallam University.

### Instruments and instrumental conditions

All mass spectrometric analyses were carried out using a Waters MALDI-QTOF Synapt G2 *Si* instrument (Waters Corporation, Manchester, UK). Data acquisition was performed within the *m/z* range 600–2000 Th in positive reflectron mode. The MALDI QTOF G2 *Si* instrument is supplied with a repetition rate Nd: YAG laser which was set to 1 kHz for these experiments. A 0.5 µL spot of saturated phosphorus red solution in ACN was used as the internal calibrant in the *m/z* range 600–2500 Th for each sample by acquiring a spectrum in the same acquisition instance as the sample. MALDI MS/MS spectra were obtained using argon as the collision gas; the trap collision energy was set at 100, laser power 300 and low mass resolution at 14.6.

LC MS/MS analyses were performed on a Xevo G2-XS QTOF (Waters Corp, Manchester, UK) equipped with an Acquity UPLC system (Waters Corp, Manchester, UK). For UPLC analysis, the injection volume was 5 µL; mobile phase **A** consisted of 0.1% formic acid in deionised water and mobile phase **B** of 0.1% formic acid in Acetonitrile (HPLC grade). Samples were run on an Acquity Ultra Performance Liquid Chromatography (UPLC) HSS T3 100 Å, 1.8 µm, 2.1 × 100 mm column at a flow rate of 0.2 mL/min starting with 3% B. The mobile phase went from 3% B to 95% B in 49 min and ramped to 98% B in the subsequent minute and was kept constant for 2 min. The mobile phase returned to 3% B in 4 min for an overall run duration of 56 min. Column temperature was set to 45 °C. MS/MS analyses were conducted in data dependent scan mode within a *m/z* range 100–1800 Th. The cone voltage was set to 40 V and Collision voltage was set to 30 V for all samples.

### Data processing

UniProt (https://www.uniprot.org/) was used to search for protein sequences of interest. In silico proteolysis with trypsin was performed by using the tool “peptide mass”. In silico peptide lists were generated by selecting “monoisotopic”, “MH^+^”, “2 missed cleavages” and “variable methionine oxidation” in the *m/z* range 600–2000 Th. Microsoft Excel tables were generated reporting the theoretical *m/z* and the sequence of peptides generated from (amongst other) haemoglobin (HB, α and β chains), erythrocyte membrane protein band 4, haptoglobin (Hpt), apolipoprotein, myoglobin, glycophorin A and albumin. Theoretical *m/z* values for the above peptides were reported for both human and the animal species investigated. Macros were generated to rapidly highlight proteotypic peptides present in the spectral peak list and those peptides identical in sequence, shared amongst the multiple species under investigation. Blood proteins were investigated from human, chicken, porcine, bovine and wild boar. UniProt does not make clear whether *sus scrofa* as taxonomy refers to the wild boar or domestic pig. Proteins from both these animals were all identified as deriving from *sus scrofa,* therefore a distinction between domestic pig and wild boar could not be made. Mass spectra were viewed both in MassLynx, (Waters Corporation, Manchester, UK) and in mMass, an open source multifunctional mass spectrometry software^11,12^, upon conversion of the spectra into .txt files. Firstly, peak centroiding was carried out in MassLynx. Spectra were then exported in mMass and only the peaks with S/N of 10 or above were labelled. Mass lists including known matrix (or matrix cluster, adduct) and trypsin autolysis *m/z* peaks were generated and used in mMass to exclude from the peak labelling irrelevant *m/z* signals. The spectral mass lists were then searched against the Excel tables. Peak assignment was automatically performed using an Excel macro. A peptide match was confirmed in mMass within a mass accuracy of 30 ppm. These signals were then ultimately checked in MassLynx and confirmed if within a relative error of 15 ppm.

MS/MS spectra opened in MassLynx were converted in .txt files and viewed in mMass for smoothing and peak labelling prior to launching an automatic Mascot MS/MS search from within the software. As search parameters, *Chordata* or *mammalia* were chosen as taxonomy when in the suspected presence of chicken (*chordata*), bovine and porcine (*mammalia*) blood respectively. Mass Tolerance was set at 40 ppm for the precursor ion and as 60 ppm for the ion fragments. MH^+^ and monoisotopic ions were selected and 2 missed cleavages and variable methionine oxidation were included in the *y*, *b, a* and *c* ion fragment search.

### Methods

#### Extraction, and proteolytic digestion and purification of blind and reference samples

The trypsin Gold and RapiGest solution was prepared just before proteolysis. RapiGest (0.1% v/v in 50 mM ammonium bicarbonate solution) was added to Trypsin Gold to reconstitute it in a 150 µg/mL solution. Stains and marks were extracted and digested by adapting a previously published method by Patel et al.^9^. Each blind stain/fingermark sample was swabbed with 70% ACN: H_2_O. The swab head was removed using scissors and transferred into an eppendorf where 1 mL of 70% ACN: H_2_O solution was added prior to sonication for 10 min. Ten µL of the 1 mL extract were added to 40 µL of 40 mM Ammonium Bicarbonate and to 9 µL of trypsin solution at a concentration of 20 µg/mL and RapiGest (0.1% v/v). The sample was then incubated for 1 h at 37 °C and the proteolytic digestion was stopped with the addition of 2 µL of 5% TFA. Digests were stored at − 80 °C until analysis. Prior to analysis samples were pre-purified using C18 ZipTips according to the standard protocol and peptides were eluted in 5 µL of 50:50 ACN: 0.1% TFA.

Commercially available intravenous animal blood samples from bovine, porcine and chicken (both with and without EDTA) were also subjected to in solution proteolytic digestion in order to obtain reference spectra. Five microliters of each blood reference sample was diluted 1 in 200 with H_2_O and 10 µL of the diluted blood were subjected to enzymatic digestion and purification protocols as described for the blind samples.

#### Matrix and application

Ten mg/mL α-CHCA in 70:30 ACN: 0.5% TFAaq was deposited by spotting 0.5 µL on top of the sample for profiling experiments.

## Results and discussion

Blind samples consisting of stains and fingermarks were prepared on aluminium slides in the following matrices: human semen, saliva and sweat, matrices unrelated to any biofluid (egg yolk, beetroot, lotions, steak sauce and ketchup), human blood, animal blood (porcine (pig and wild boar), bovine and avian (chicken)). All of the samples were supplied either as unenhanced or enhanced using three blood enhancement techniques (BET) such namely Acid Black 1 (AB-1), Acid Yellow 7 (AY-7) and Leucocrystal Violet (LCV). To the authors’ knowledge this is the first study of its kind to address validation of a technique for blood detection and provenance determination using blind samples, and of considerable diversity, in combination with the prior application of BET.

Within the selected pool of animal species and biofluids, the data acquired from the blind samples can theoretically enable five levels of identification (ID levels I–V) namely (I) confirmation of presence/absence of blood; (II) determination of blood provenance (human or animal); (III) determination of blood provenance at animal species level; (IV) determination of the presence of biofluid other than blood; ((V) determination of the type of biofluid. This study focussed on the first three levels of identification. However, during the study, semen biomarkers were also identified thus tapping into ID levels IV–V.

### Development of the interpretative strategy

**Figure 1:**
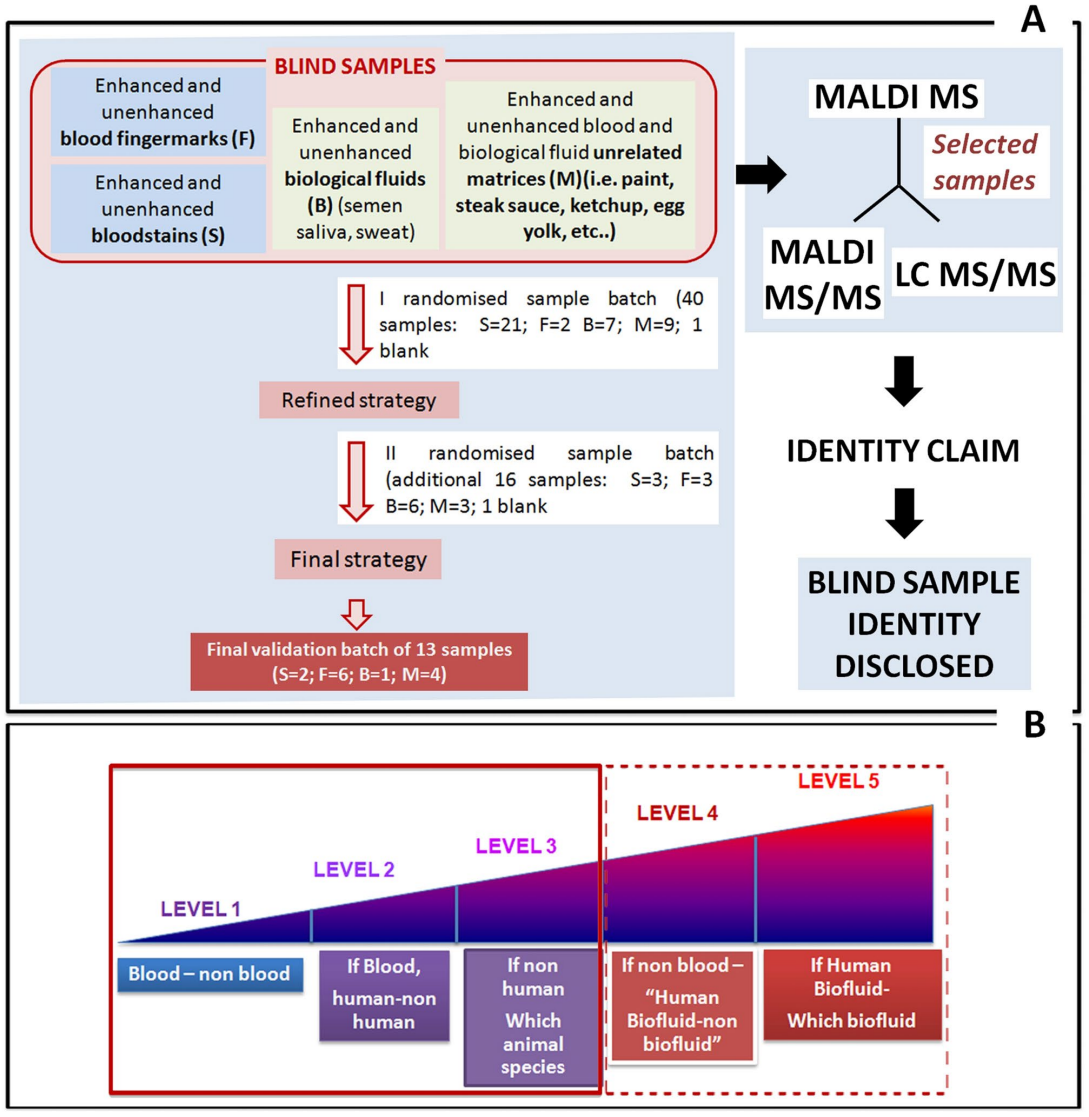
Blind samples investigated and general experimental and analytical approach. (**A**) shows the subdivision of the blind samples and their investigation in a stepwise approach within a refining strategy validated through a final subset of 13 blind samples. Analyses were performed by MALDI MS with some selected samples submitted to MALDI MS/MS and/or LC MS/MS for protein identification/identity confirmation. (**B**) shows the five levels of identification theoretically permitted by the data. Of these levels, this study pursued the first three (continuous line) and generated some knowledge towards levels 4 and 5 (dotted line).

The main interpretative hypothesis was that by targeting proteins specific to human or animal blood, it was possible to both detect and source attribute this biofluid. MALDI MS, MALDI MS/MS and LC MS/MS were employed for a selected sample subset to both determine and confirm the identity of relevant ion signals. Once the analysts had made an identity claim on the sample analysed, its true identity was disclosed to assess effectiveness of the interpretative strategy. Figure 1 summarises the type of samples investigated and the general strategic approach. Retrospective analyses of samples that had been incorrectly classified allowed for strategy refinement in an iterative manner. This refinement included the re-definition of the S/N threshold and the mass accuracy for peak picking (the latter based in part on the instrumental calibration and in part on average instrumental performance) which were eventually set at 10 and 15 ppm respectively. For animal blood attribution, sample preparation was modified with respect to the initial experimental protocol by introducing a pre-purification step using C18 ZipTips after proteolysis. Sample purification led to a better S/N ratio and more peptide ion populated spectra when in the presence of animal blood. Initially, for animal blood attribution, in addition to identifying the frequency of the putative presence of proteotypic peptides across the animal species investigated (within the subset of blood proteins taken in consideration), the frequency of putatively detected *shared* peptides per species was used as an additional interpretative criterion. However, this criterion confounded data interpretation leading to the wrong claim and was therefore discounted.

### ID level I: detection of blood

Initially, detection of blood was based on the detection of the two most abundant haemoglobin (HB) peptides at *m/z* 1274.725 (βHB LLVVYPWTQR) and at *m/z* 1529.734 (αHB VGGHAAEYGAEALER). Within the system under investigation, these peptides are shared between human, porcine and bovine species only; therefore, if chicken blood was present, this would go undetected. However, an unexpected chicken blood marker was later detected (see “ID level III: influence of blood collection method and animal blood species attribution” section), thus permitting ID Level I when in presence of any of the blood sources investigated*.*

### ID level II: discrimination between human and animal blood

The distinction between human and animal blood was not straightforward. The two most abundant peptide markers at *m/z* 1274.725 and *m/z* 1529.734 were used again as positive blood markers as a starting point.

The βHB peptide at *m/z* 1274.725, despite being clearly at its highest intensity in human blood mass spectra, is also shared with porcine and bovine blood. Similarly, the αHB at *m/z* 1529.734 is present in both human and bovine blood. Therefore, the simultaneous presence of both peptides could only mean that the sample did contain blood and could be of either human or bovine origin (in this study, where the blood was known to have single provenance).

To determine whether the blood was of human versus animal (bovine) origin, identification initially relied on the detection of human blood protein derived proteotypic peptides such as that at *m/z* 932.520 (βHB peptide), *m/z* 1087.553 (EPB42, UniProtKB–P16452) and 1378.694 (Hpt UniProtKB–P00738). However, these additional ion signals were only occasionally detected and often of a low intensity and resolution or borderline acceptable mass accuracy. Therefore, eventually, an ID Level II claim was made and human blood was identified if both of the two signals at *m/z* 1274.725 and 1529.734 were present, with the caveat that there might have been instances of false (human blood) positives considering that these peptides are found in bovine HB amino acid sequence too. In practice, it has been verified that in all the samples examined, both of the above signals are present together only in human blood. Therefore, this ID Level II criterion was brought forward for interpreting the final set of 13 pre-validation samples (see “Analysis of the final validation set of blind samples” section). The reliance on the simultaneous detection of the signals at *m/z* 1274.725 and *m/z* 1529.734 for human blood resulted in a 1/40 false positive for human blood (Sample 1 S was bovine blood) (Table 1).

**Table 1:** Initial MALDI MS proteomic analysis of 40 blind samples randomly selected and in order of analysis.

Sample no	BET & reaction	I ID level (blood?)	II ID level (if blood, human/animal)	III ID level (animal species?)	True identity	Correct claim?
1 S	None	Yes	Human	N/A	Bovine	No
2 S	None	Yes	Human	N/A	Human	Yes
3 S	None	Yes	Human	N/A	Human	Yes
5 S	AB-1 +	Yes	Animal	Bovine	Bovine	Yes
6 S	None	Yes	Human	N/A	Human	Yes
7 S	None	Yes	Inconclusive	Inconclusive	Porcine	No
12 S	None	No	N/A	N/A	Sweat	Yes
13 S	LCV+	No	N/A	N/A	Porcine	No
14 S	AB-1 +	No	N/A	N/A	Human	No
16 S	None	Yes	Human	N/A	Human	Yes
17 S	None	Yes	Human	N/A	Human	Yes
18 S	None	Yes	Animal	Porcine	Wild boar	Yes
26 S	LCV−	No	N/A	N/A	Saliva	Yes
27 S	None	No	N/A	N/A	Semen	Yes
28 S	None	No	N/A	N/A	Bovine	No
29 S	AY-7 +	No	N/A	N/A	Chicken	No
30 S	AB-1 +	No	N/A	N/A	Egg yolk	Yes
31 S	LCV+	Yes	Human	N/A	Human	Yes
34 S	LCV+	Yes	Human	N/A	Human + EDTA	Yes
35 S	None	No	N/A	N/A	Chicken	No
36 S	None	No	N/A	N/A	Ketchup	Yes
37 S	None	Yes	Human	N/A	Human	Yes
40 S	None	No	N/A	N/A	Sweat	Yes
41 S	AB-1 +	No	N/A	N/A	Ketchup	Yes
49 S	AB-1 +	No	N/A	N/A	Saliva	Yes
53 S	None	Yes	Human	N/A	Human + EDTA	Yes
56 S	None	No	N/A	N/A	Porcine	No
57 S	None	No	N/A	N/A	Paint	Yes
59 S	None	Yes	Human	N/A	Human	Yes
60 S	AY-7 +	No	N/A	N/A	Saliva	Yes
61 S	None	Yes	Human	N/A	Human	Yes
63 S	LCV− faint	No	N/A	N/A	Chicken	No
78 S	None	No	N/A	N/A	Lotion gold bond	Yes
79 S	None	No	N/A	N/A	Blank	Yes
122 F	AY-7 +	Yes	Human	N/A	Human	Yes
141 F	AB+ (spotty)	No	N/A	N/A	Ketchup	Yes
160 F	AY-7 −	No	N/A	N/A	Saliva	Yes
162 F	AB-1 +	Yes	Human	N/A	Human	Yes
165 F	AY-7 +	No	N/A	N/A	Egg white	Yes
175 F	LCV−	No	N/A	N/A	Egg white	Yes

S (stain), F (fingermark). BET indicates “blood enhancement technique” and the corresponding column shows “none” for none applied or reports the name of the technique with the enhancement result; “−” indicates no enhancement whereas “+” indicates enhancement. AB-1 (Acid Black 1); LCV (Leucocrystal Violet); AY-7 (Acid Yellow 7).

Table 1 also shows that out of 15 human samples, only sample 14 S (in which the signals at nominal *m/z* 1275 and 1530 were absent) resulted in a false negative for human blood. Other *m/z* proteotypic peptides for human blood previously identified were also absent. A new protein digest was prepared from sample 14 S extract yielding the same results.

As the original sample was no longer available and given that this is the only false negative for human blood, it is speculated that sample mislabelling occurred (a different analyst prepared the sample at the time). As Table 1 shows, the method also works for human blood mixed with EDTA (chemical agent preventing blood coagulation). Furthermore, correct human blood identifications were made compatibly with the prior application of blood enhancement techniques (LCV, AY-7 and AB-1). These tests correctly indicated the presence of blood but it is important to bear in mind that they cannot discriminate between blood of human and animal provenance.

When in the presence of matrices unrelated to blood or to any biofluid (such as ketchup, egg yolk, paint etc.), the samples were correctly classified, using the MALDI MS based strategy, as “non-blood” (9/9 correct classification). Interestingly, contrary to MALDI MS results, the presumptive test incorrectly indicated blood presence in a few instances (e.g. sample 41—ketchup; sample 30—egg yolk; sample 165—egg white).

#### ID level III: influence of blood collection method and animal blood species attribution

**Figure 2:**
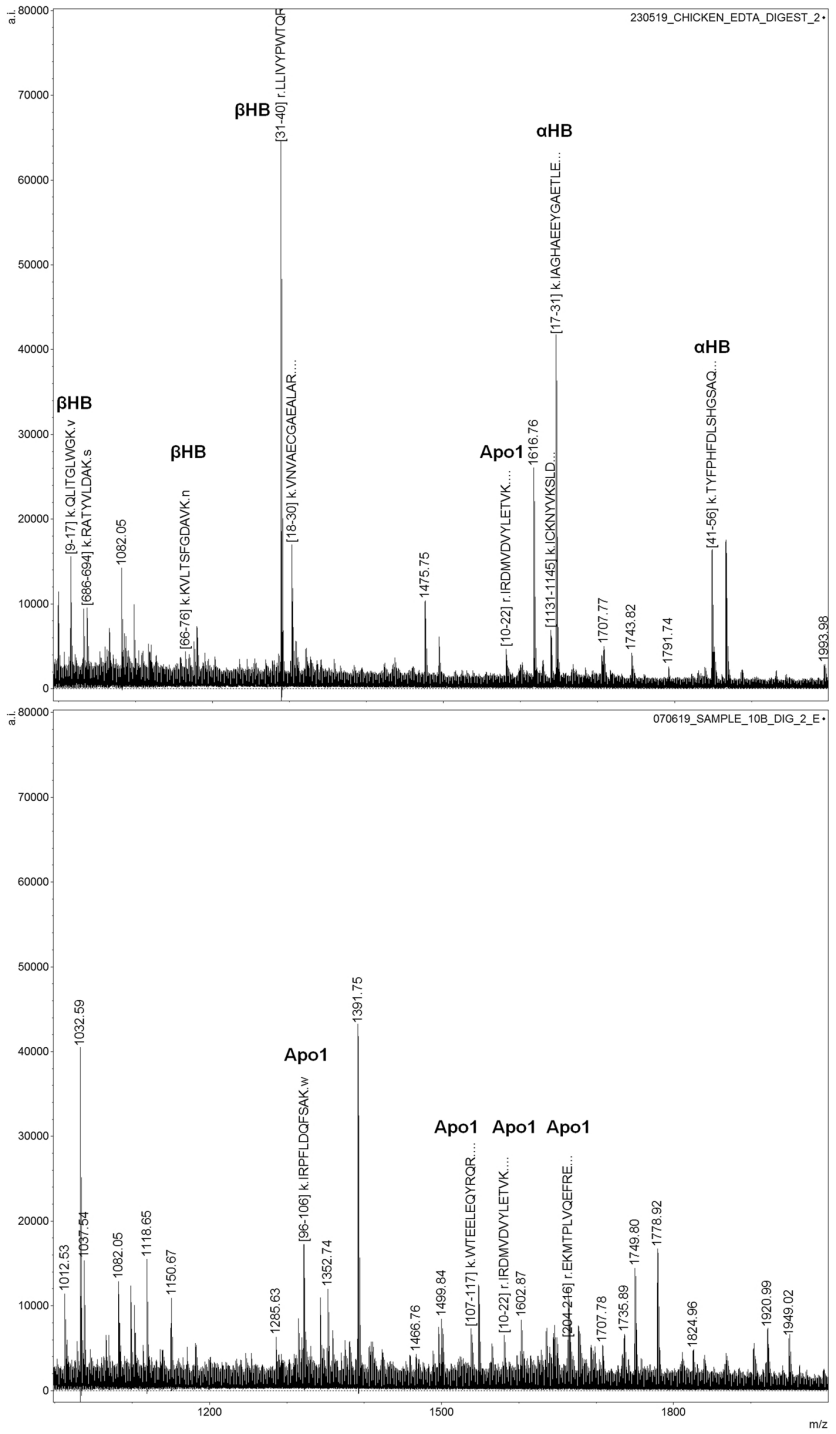
MALDI MS spectra of commercially available chicken blood (top panel) and of blind sample 10 (chicken bloodstain, bottom panel) showing overall different spectral profiles.

The adoption of HB and other animal blood specific protein markers (through proteotypic peptides) largely failed to discriminate the provenance of animal blood. Table 1 shows that for 7/9 animal blood samples, the MALDI MS based strategy yielded an incorrect “non-blood” claim with 3/3 false negatives for chicken blood, 3/4 false negative for porcine blood and 2/3 false negatives for bovine blood. In order to understand why animal blood classification was unsuccessful, blood reference samples for bovine, porcine and chicken were investigated to determine the peptide targets to search for within the animal blood blind sample spectra. It was quickly determined that, for all the species, the commercially available blood reference spectra were very different from the corresponding blind animal sample spectra. Figure 2 shows an example comparing the MALDI MS spectra from commercially available chicken blood and from the blind chicken blood sample 10. As it can be seen, HB ion signals at *m/z* 999.487, 1036.561, 1164.648, 1288.736, 1302.645, 2226.129 (βHB, UniProtKB–P02112) and at *m/z* 1645.776, 1847.900, 2121.142 and 2249.224 (αHB, UniProtKB–P01994) could only be putatively observed for the reference blood. At this stage it was disclosed that, while human blood was collected through phlebotomy, the animal blood collected for the blind study was obtained from a butcher in the US by harvesting any volume of blood that had collected in the chest cavity which then was pooled with a large volume syringe (one per animal).

The collection of chicken blood was reported to be the most challenging due to significantly less blood present and the quick dilution with water during the cleaning/processing of the animal. This could explain the lack of detection of HB signals in the blind sample 10. At this stage, only the peptides at *m/z* 1321.746, 1537.754, 1580.817, 1662.858, could be putatively assigned in the blind sample 10 spectrum and were attributed to apolipoprotein 1 (ApoA1, UniProtKB–P08250). Of these 4 assignments, only the ApoA1 signal at *m/z* 1580.817 (1–22, IRDMVDVYLETVK) was found in both sample 10 and the blood reference spectrum.

Notably the most intense ion signals at *m/z* 1391.746, and 1749.798 and 1778.923 could not be assigned to any of the most abundant/blood specific proteins under investigation nor could a Mascot search identify these peaks.

The same type of spectral comparison is reported in Supplementary Fig. S1 for bovine (A) and porcine (B) blood. The blind samples exhibited a much greater complexity in terms of ion population.

Furthermore, porcine and bovine HB signals, clearly present in the reference spectra (from commercially available blood) could not be detected in the corresponding blind sample spectra. The reference blood had been collected for all animals through an incision of the jugular vein with the blood subsequently stored in EDTA. This would explain why HB peptide signals are the most intense in the reference blood spectra.

As blind samples exhibited a different mass spectral profile from the corresponding intravenous blood, it was hypothesised that a different method of collecting blood, closer to how the blood samples were prepared, could be more suitable than intravenous blood to act as a reference blood. Consequently, it was investigated whether the blood collected from packaged meat and blood residues originating from butcher prepared meat had mass spectral profiles superimposable with those from the blind samples or the reference spectra. This investigation was crucial to the ability to discriminate animal species and it became even more interesting because, if the spectra were different, then the difference would be strictly correlated to the way in which blood was harvested.

For blood detection studies, this circumstance would imply that it is incorrect to just target the most abundant proteins that one would normally expect to find in blood. If different blood protein compositions are possible, these different “systems” need to be understood. Wounded animals perhaps resemble the closer scenario to intravenous blood; blood traces from touching the meat prepared directly from a butcher is possibly a more common scenario at crime scenes and, with the widespread purchasing and handling of packaged meat, bloodstains or blood fingerprints could also derive from this form of animal blood contamination.

##### Determination of a chicken blood biomarker

**Figure 3:**
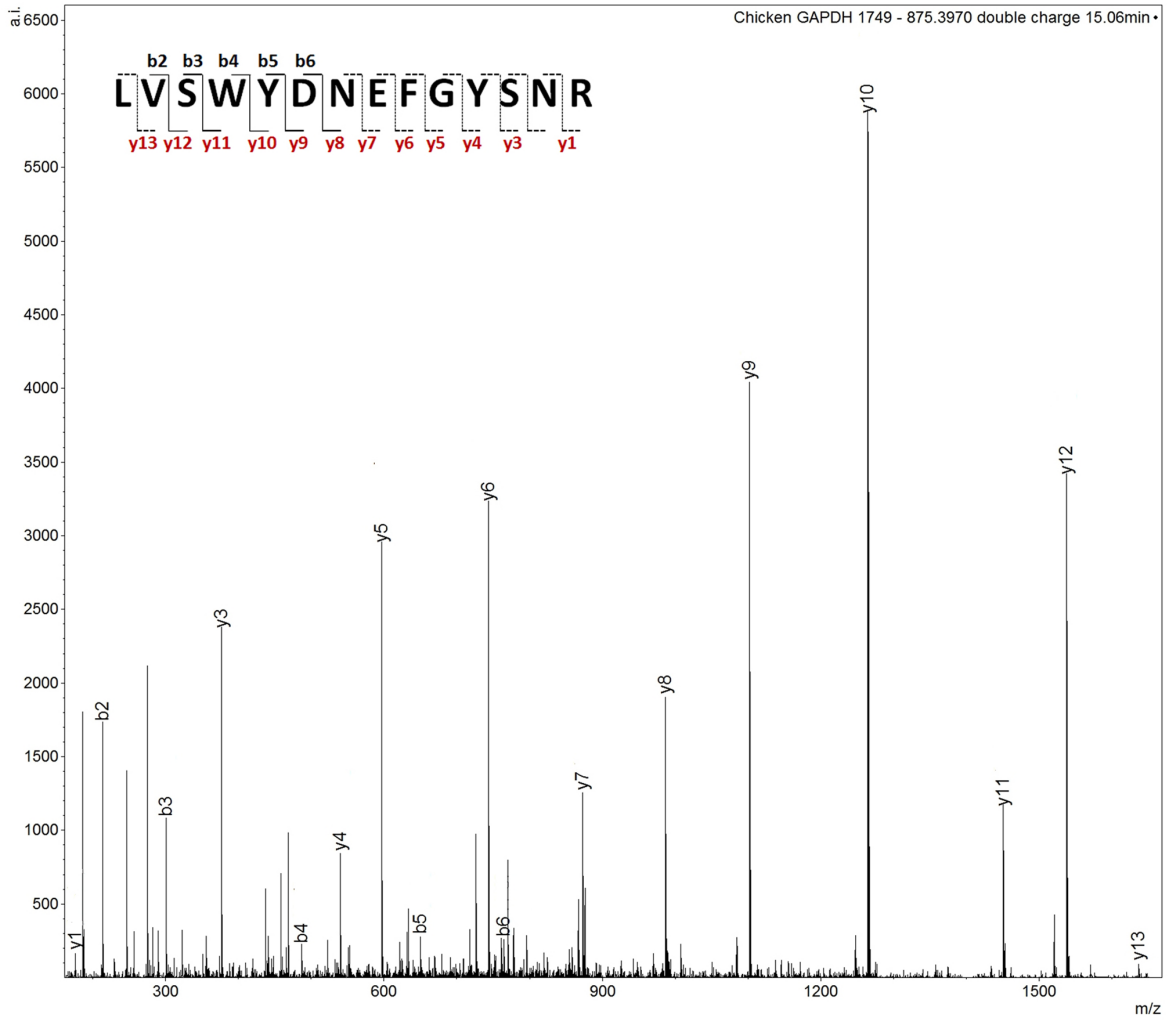
LC MS/MS spectrum of the doubly charged ion at *m/z* 875.397 in sample 35 S. This spectrum yielded the identification of chicken GAPDH with a Mascot score of 92. The fragment ions not annotated on the peptide sequence in the figure indicate that they have not been detected.

As mentioned above, for chicken blood, the signal at *m/z* 1749.798 detected in the blind sample 10 was one of the most intense unassigned signals. This ion signal was also present in packaged and butcher freshly prepared meat but not in the intravenous blood. Initially, a MALDI MS/MS analysis was performed on this ion at nominal *m/z* 1750 in the freshly prepared meat, the packaged meat and blind samples. All but the blind sample yielded the identification of glyceraldehyde 3-phosphate dehydrogenase (GAPDH) (UniProtKB P00356), through the peptide L**V**SWYDNEFGYSNR (theoretical *m/z* 1749.787), with a Mascot score of 117 and 62 respectively which are of statistical significance. The subsequent use of LC MS/MS enabled confirmation of GAPDH within the blind sample 35 S (as sample 10 S was no longer available) (Fig. 3), through the doubly charged ion at *m/z* 835.397 eluting at 15.06 min, with a Mascot score of 92.

Table 2 summarises the MS/MS GAPDH identifications from the three samples analysed.

**Table 2:** Summary of selected sample submitted to MS/MS analysis and yielding identification/confirmation identity of a chicken blood biomarker (GAPDH).

Ion signal *m/z* (Th)	Analysis	Mascot Score	ID protein and UniProt accession number	Sequence	Ion fragments detected
**Chicken packaged meat**					
1749.855	MALDI MS/MS	62	GAPDH–P00356	LVSWYDNEFGYSNR	y1–y12; b8–b9
**Chicken butcher freshly prepared meat**					
1749.793	MALDI MS/MS	117	GAPDH—P00356	LVSWYDNEFGYSNR	y1–y12; b6–b8
**Chicken sample 35 S**					
875.397	LC MS/MS	92	GAPDH—P00356	LVSWYDNEFGYSNR	y1, y3–13; b2–b6

Following the peptide identification at nominal *m/z* 1750, other GAPDH signals were searched for in the spectra of both sample 35 S and 10 S (chicken blood) and the additional GAPDH signals at a nominal *m/z* of 795, 805, 1033, 1359 and 1646 were assigned with a mass accuracy ranging between 13.2 and 3.6 ppm.

##### Determination of bovine and porcine blood biomarkers

**Figure 4:**
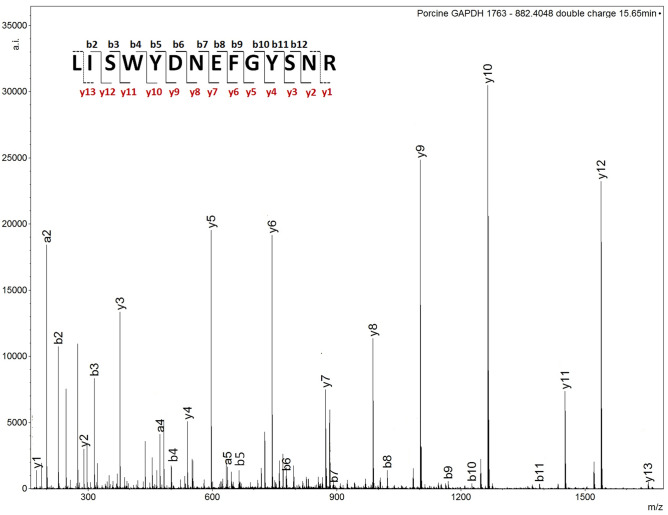
Annotated LC MS/MS spectrum of *m/z* 882.405 from sample 7 S confirming the presence of porcine GAPDH. Missing annotation on the peptide sequence denotes lack of detection of that particular b/y ion fragment.

*Glyceraldehyde-3 phosphate-deydrogenase (GAPDH)* The signal at *m/z* 1749.787 was absent in bovine and porcine blind blood samples. However, a signal at *m/z* 1763.802 was consistently present in the bovine and porcine blind sample spectra but absent in the corresponding intravenous blood. MALDI MS/MS analysis and Mascot searches were performed on this ion at nominal *m/z* 1764 for porcine sample 7 S and packaged meat, yielding the identification of porcine GAPDH (UniProtKB–P00355) with a statistically significant Mascot score of 69 and 122 respectively, through the peptide of sequence L**I**SWYDNEFGYSNR. This sequence differs from that found in the chicken GAPDH protein by an isoleucine replacing a valine (mass difference of 14 units) in the second position of the peptide. The presence of GAPDH in sample 7 S was additionally confirmed with a Mascot score of 93, through LC MS/MS of the doubly charged ion at *m/z* 882.405 (Fig. 4) eluting at 15.65 min.

Bovine GAPDH (UniProtKB-P10096) was confirmed through LC MS/MS of blind bovine sample 38 S with a MASCOT score of 100 by fragmenting the doubly charged ion at *m/z* 882.405.

As for the chicken blood, the blood spectral profiles for the blind bovine and porcine samples (38 S and 7 S respectively) reported in Fig. S1 were re-investigated. With reference to these spectra, GAPDH was putatively detected via multiple peptides in both the bovine and porcine blind samples but not in the corresponding intravenous blood. However, the only ion signal consistently present across all of the bovine and porcine samples analysed to that point was that at nominal *m/z* 1764.

GAPDH is normally used as a housekeeping gene or ‘internal control in experiments’ due to its relatively constant expression at high levels in skeletal muscle under changing conditions^13^ (in chicken, GAPDH is reported to have the highest expression in skeletal muscle). The expression of this protein explains why these signals were only detected in the blind samples and not in the intravenous blood.

Therefore, ultimately, the hypothesis that blood has a different protein profile, depending on how “blood was generated/collected” has been verified, at least with reference to the way the blood was collected for the blind animal sample preparation and the animal intravenous blood.

Table 3 reports and summarises the selected samples submitted to MS/MS and yielding GAPDH biomarker identification of porcine and bovine blood.

**Table 3:** Summary of selected sample submitted to MS/MS analysis and yielding identification/confirmation identity of porcine and bovine blood biomarkers.

Sample	Ion signal *m/z* (Th)	Analysis	Mascot Score	Protein	Sequence	Ion fragments detected
**Porcine blood**						
7 S	1763.811	MALDI MS/MS	69	GAPDH	L**I**SWYDNEFGYSNR	y1–y9; b3, b8
7 S	882.405	LC MS/MS	93	GAPDH	L**I**SWYDNEFGYSNR	y1–y12; b2–11; a2, a4–a5, a8, a11
Packaged meat	1763.786	MALDI MS/MS	122	GAPDH	L**I**SWYDNEFGYSNR	y1–y12; b2–b6, b8–b11; a2
**Bovine blood**						
38 S	882.405	LC MS/MS	100	GAPDH	L**I**SWYDNEFGYSNR	y2–y12; b2–b3, b5, b8; a2, a4
38 S	796.921	LC MS/MS	93	Myoglobin	VEADVAGHGQEVLIR	y1, y7–13; b2, b4–b5, b11–b12, b14; a2
1 S	1592.822	MALDI MS/MS	58	Myoglobin	VEADVAGHGQEVLIR	y7–y8, y10–y11,y13; b8–b8,b11,b13; a8, a12; c9

The identification of the signals at nominal *m/z* 1750 and 1764 permitted a data interpretation strategy refinement; when blood presence is confirmed and human blood is excluded, the presence of the ion at nominal *m/z* 1750 indicates presence of chicken blood via the protein GAPDH. Should the ion at nominal *m/z* 1750 be absent, the GADPH peptide at *m/z* 1763.802 confirms the presence of animal blood but cannot distinguish between porcine and bovine blood.

**Figure 5:**
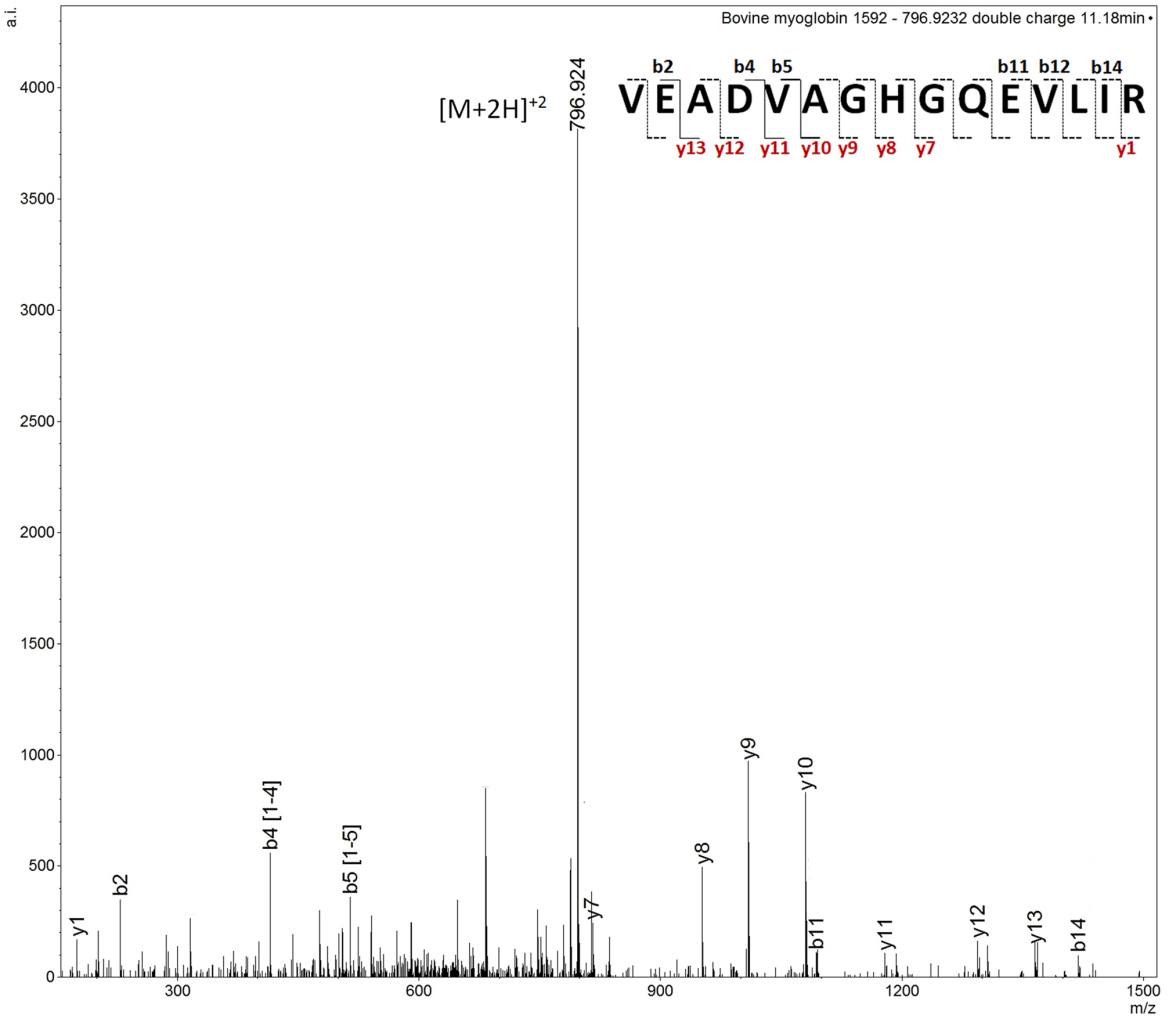
Annotated LC MS/MS spectrum of doubly charged ion at *m/z* 796.924 from sample 38 S confirming the presence of porcine GAPDH. Missing annotation on the peptide sequence denotes lack of detection of that particular b/y ion fragment.

*Myoglobin-* Further investigation into the spectra of the blind bovine blood samples that had been incorrectly classified (Table 1) allowed for the putative identification of other bovine blood markers. Bovine blood exhibited the presence of the signals at *m/z* 1669.837 and *m/z* 1592.822 which were putatively attributed to myoglobin (UniProtKB–P02192), initially on the basis of their *m/z* and their mass accuracy (< 15 ppm). Myoglobin peptide at nominal *m/z* 1593 was confirmed (VEADVAGHGQEVLIR) in the blind bovine blood sample 38 S through LC MS/MS by fragmenting the doubly charged ion signal at *m/z* 796.924 eluting at 11.18 min, with a MASCOT score of 93 (Fig. 5).

This identification was also confirmed in sample 1 S (previously incorrectly classified) via MALDI MS/MS of the ion at *m/z* 1592.829, with a statistically significant Mascot score of 58 (Table 3). It was not possible however, to assign definitively the peptide at *m/z* 1669.837 by either MALDI MS/MS or LC MS/MS analysis due to an insufficient number of ion fragments. However, this signal was still used as a putative identification for bovine myoglobin. Although the signal at nominal *m/z* 1593 appears in all of the bovine samples, that at nominal *m/z* 1670 does not. The former ion signal shares the same sequence in porcine myoglobin though it is never detected in the porcine blind samples. Another signal at nominal *m/z* 649 was identified as porcine myoglobin (UniProtKB–P02189) on the basis of the *m/*z and mass accuracy (< 10 ppm). This signal was present in all but one porcine blind samples analysed.

Therefore: (i) the presence of bovine blood was claimed if markers at nominal *m/z* 1764, 1593 and/or 1670 were present (within the set mass accuracy); (ii) the presence of porcine blood was claimed if the signal at a nominal *m/z* 1764 was present and signals at nominal *m/z* 1593 and/or 1670 were absent. Confidence in making this claim was increased if the signal at nominal *m/z* 649 was also present.

Following the identification of myoglobin through the peptide at nominal *m/z* 1593, this protein was also retrospectively identified in bovine sample 38 S (but not in the corresponding intravenous blood) though multiple peptides with a mass accuracy ranging between − 11.2 and 2.5 ppm.

Supplementary Table S1 summarises the identifications of haemoglobin, GAPDH and myoglobin biomarkers in blind samples 7 S (porcine blood) and sample 38 S (bovine blood) versus the corresponding intravenous reference blood showing marked differences in the presence of these proteins. The presence and nature of GAPDH, and myoglobin in the blind samples aligns with the location from which blood was harvested.

Within the "system" investigated, altogether these results indicate the opportunity of performing further source attribution (ID Level III) by differentiating chicken, bovine and porcine blood through a refined strategy.

### Identification of semen: a glimpse into ID levels IV and V

A further sample had been incorrectly classified as human blood. This was one of the earliest blind samples analysed and the claim was incorrectly based on the presence of only one signal, the αHB peptide at *m/z* 1529.734 (mass accuracy 0 ppm).

**Figure 6:**
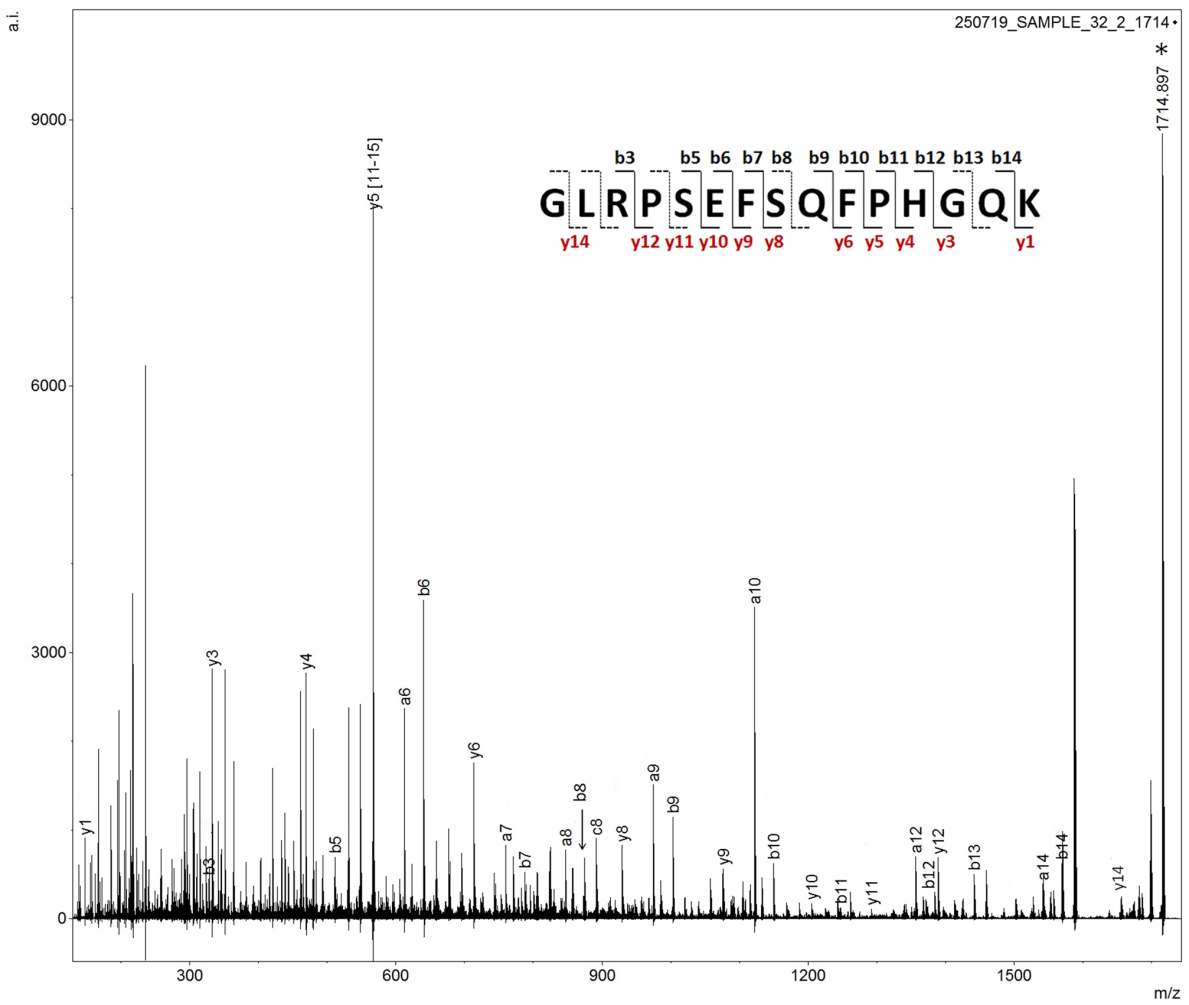
MS/MS spectrum and ion fragment annotation of SEM-1 detected in sample 32. b and y ion are annotated and also shown on the peptide sequence; a and c fragment ions are only annotated in the spectrum. Missing annotation on the peptide sequence denotes lack of detection of that particular b/y ion fragment.

Upon disclosure of the sample identity, the spectrum was inspected more carefully and an intense signal at *m/z* 1714.849 was observed. MALDI MS/MS analysis was performed on this precursor ion and a Mascot search was launched. Through this search semenogelin-1 (SEM-1 UniProtKB–P04279) was identified through the peptide sequence GLRPSEFSQFPHGQK, with a statistically significant score of 99. The MS/MS spectral annotation of the ion fragments is shown in Fig. 6.

SEM-1 is produced in the seminal vesicles^14^ and it is abundant in semen. Semenogelin was also identified by Iliano et al. by LC MS/MS^4^ and it is the target for a semen confirmatory test employing a lateral flow immunochromatographic test strip containing colloidal gold-conjugated anti-human semenogelic monoclonal antibodies^15^ (Rapid Stain Identification (RSID)).

Additional ion signals were putatively assigned to this protein at *m/z* 1444.764, 1501.744 and 1801.918 with a mass accuracy of 6.6, 0.5 and 13.1 ppm respectively (Supplementary Fig. S2).

Encouraged by these results, a thorough search of other semen specific proteins was performed on the basis of the literature available and Semenogelin-2 (SEM-2, UniProtKB–P04279) was also putatively detected (Supplementary Fig. S2) (*m/z* 1883.936, mass accuracy 0.3 ppm; *m/z* 1444.764, shared with SEM-1, mass accuracy 6.6 ppm; *m/z* 1554.779, mass accuracy 8.6 ppm). SEM-2 has additionally been reported as a protein biomarker for semen detection through mass spectrometric based techniques and also using miRNA^16–18^. Of these ion signals, those at nominal *m/z* 1445 (SEM-1/SEM-2) and 1555 (SEM-2), were consistently found in the semen samples analysed; therefore these markers were also implemented for the rapid identification of remaining semen samples in the study. The putative presence of the signals at *m/z* 1529.734 (αHB) and 1314.682 (βHB) does not allow for the exclusion of the (weak) co-presence of blood within semen. This instance was important for the consideration that, within real crime scenes, it is possible that blood and semen may both be present in a stain; therefore confirmatory MS/MS analyses would be needed given that the finding would be of significant relevance to an investigation.

Upon retrieval of the SEM-1 marker at nominal *m/z* 1715, subsequently semen samples (blind to the analyst) were not only dismissed as blood but also correctly identified as semen (Table 4).

**Table 4:** Analysis and identification of semen blind samples.

Sample no.	BET	I ID Level (Blood?)	II ID level (if blood, human/animal	III ID level (which animal species?)	True identity	Correct claim?
32 S	LCV −	Yes	Human	N/A	Semen	No
**Following retrieval of semenogelin-1 marker at nominal m/z 1715**						
27 S	None	No	N/A	N/A	Semen	Yes
58 S	AB-1 +	No	N/A	N/A	Semen	Yes
132 F	LCV (− 1 area/residue)	No	N/A	N/A	Semen	Yes
158 F	AB-1 +	No	N/A	N/A	Semen	Yes

S (stain), F (fingermark). BET indicates “blood enhancement technique” and the corresponding column shows “none” for none applied or reports the name of the technique with the enhancement result; “−” indicates no enhancement whereas “+” indicates enhancement. AB-1 (Acid Black 1); LCV (Leucocrystal Violet).

Semen was correctly classified even when the sample was pre-enhanced with Acid Black 1 (AB-1) or Leucocrystal violet (LCV). Crucially, where AB-1 gave a positive reaction (samples 58 S and 158 F), MALDI analyses correctly refuted the results of the presumptive test identifying semen instead.

**Figure 7:**
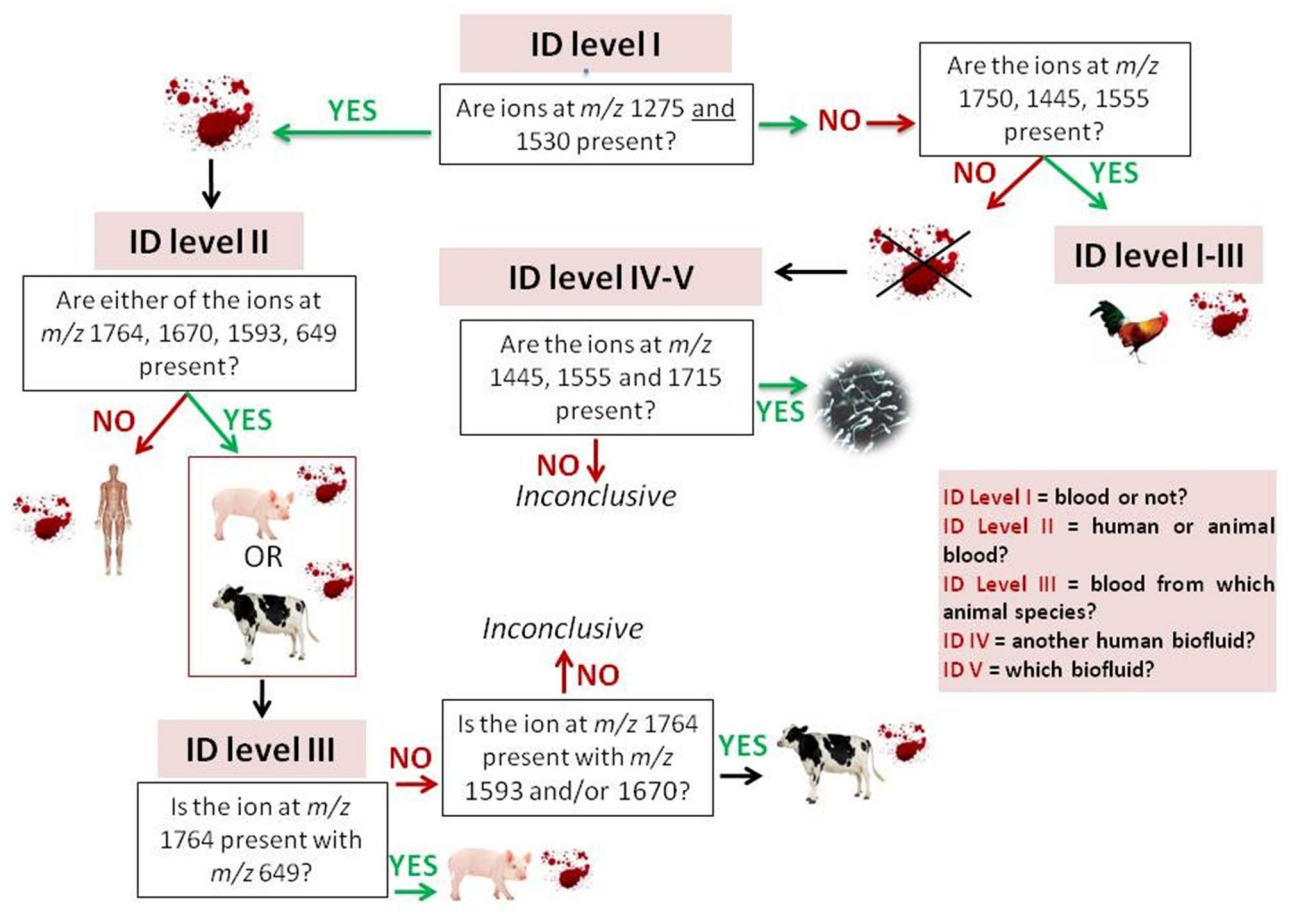
Refined blind sample spectra data interpretation strategy. This strategy enables the determination of the ID Levels I–III of animal provenance down to species as well as the determination of the presence of semen (ID Level IV and V). The *m/z* values are nominal. Their presence is verified with a mass accuracy < 15 ppm.

Figure 7 reports the interpretative strategy that was finalised to identify blood samples (from human and animal species) and semen. This strategy was used for revisiting some of the samples incorrectly identified and 11 additional blind samples as described in the following section.

### Blind sample cohort summary of results after identification of animal blood markers and partial re-visitation of some samples

Following the identification of bovine/porcine and chicken blood markers, some of the samples incorrectly classified were either re-acquired or re-prepared (extraction, proteolytic digestion and purification). Also, 11 new blind samples were analysed encompassing a mixture of human and animal blood, biofluids and non-biofluids.

Table 6 reports the identification results for a total of 56 samples including the 44 reported in Tables 1 and 4 (with some of the samples being re-visited as explained above) and the additional 11 samples. As it can be seen from Table 3, a 0% false positive rate for human blood was obtained.

One more false negative for human blood occurred for sample 170 F (total 2/13 false negatives), the spectrum of which exhibited none of the ion signals characteristic of blood. A case of mislabelling is still plausible. However, upon disclosure of sample identity, it was revealed that it was a human blood “trace”, meaning that it originated from the last mark of a depletion series deposited from a nearly dry and exhausted blood source on the fingertip.

The deposited mark was barely visible (as visible as a latent mark) and it is possible that the blood transfer was so minimal that the instrumental sensitivity was insufficient.

When blind non biofluid-related samples were analysed (beetroot juice, paint, ketchup), again a 0% false positive rate for blood was achieved (0/12 samples). When in the presence of biofluids, semen was correctly identified every time (100% correct identification rate) and a 0% false positive rate for blood was achieved overall (0/13 samples).

When in the presence of animal blood, whilst prior to discovery of the GAPDH and myoglobin marker, 7/9 samples were incorrectly classified as “non blood”, after deployment of the refined strategy (Fig. 7) the false negative rate decreased from 77.7% (7/9 samples) to 6.7% (1/15 samples). In particular, before strategy refinement a false negative rate of 100% (3/3 samples), 75% (3/4 samples) and 66.7% (2/3 samples) was yielded for chicken, porcine and bovine respectively. After strategy refinement, the false negative rate decreased to 0% (0/5 samples), 16.7% (1/6 samples) and 0% (0/4 samples) for chicken, porcine and bovine respectively. The additional new 11 samples analysed after strategy refinement shown in Table 5 (in bold) were all correctly classified, whether enhanced or not.

**Table 5:** MALDI MS proteomic analysis and identification after strategy refinement encompassing re-visitation of some previously incorrectly classified samples.

Sample no.	BET	I ID level (blood?)	II ID level (if blood, human/not)	III ID level (which animal species?)	True identity	Revisited?	Correct claim?
1 S	None	Yes	Animal	Bovine	Bovine	Yes	Yes
2 S	None	Yes	Yes	N/A	Human blood		Yes
3 S	None	Yes	Yes	N/A	Human blood		Yes
5 S	AB- +	Yes	Animal	Bovine	Bovine		Yes
6 S	None	Yes	Human	NA	Human	Yes	Yes
7 S	None	Yes	Inconclusive	Inconclusive	Porcine	Yes	Yes
12 S	None	No	N/A	N/A	Sweat		Yes
13 S	LCV+	No	N/A	N/A	Porcine	Yes	Yes
14 S*	AB-1 +	No	N/A	N/A	Human		No
16 S	None	Yes	Human	N/A	Human		Yes
17 S	None	Yes	Yes	N/A	Human blood		Yes
18 S	None	Yes	ANIMAL	Porcine	Wild boar		Yes
26 S	LCV−	No	N/A	N/A	Saliva		Yes
27 S	None	No	N/A	N/A	Semen		Yes
28 S	None	No	N/A	N/A	Bovine	Yes	Yes
29 S	AY-7 +	No	N/A	N/A	Chicken	Yes	Yes
30 S	AB-1 +	No	N/A	N/A	Egg yolk		Yes
31 S	LCV+	Yes	Human	N/A	Human		Yes
32 S	LCV−	No	N/A	N/A	Semen	Yes	Yes
34 S	LCV+	Yes	Human	N/A	Human + EDTA		Yes
35 S	None	No	N/A	N/A	Chicken	Yes	Yes
36 S	None	No	N/A	N/A	Ketchup		Yes
37 S	None	Yes	Human	N/A	Human		Yes
40 F	None	No	N/A	N/A	Sweat		Yes
41 S	AB-1 +	No	N/A	N/A	Ketchup		Yes
49 S	AB-1 +	No	N/A	N/A	Saliva		Yes
53 S	None	Yes	Human	N/A	Human blood + EDTA		Yes
56 S	None	No	N/A	N/A	Porcine	Yes	Yes
57 S	None	No	N/A	N/A	Paint		Yes
58 S	AB-1 +	No	N/A	N/A	Semen		Yes
59 S	None	Yes	Human	N/A	Human		Yes
60 S	AY-7 +	No	N/A	N/A	Saliva		Yes
61 S	None	Yes	Human	N/A	Human		Yes
63 S	LCV− faint	No	N/A	N/A	Chicken	Yes	Yes
78 S	None	No	N/A	N/A	Lotion gold bond		Yes
79 S	None	No	N/A	N/A	Blank	Yes	Yes
122 F	AY-7 +	Yes	Human	N/A	Human blood		Yes
132 F	LCV (− 1 area/residue)	No	N/A	N/A	Semen		Yes
141 F	AB-1 + (spotty)	No	N/A	N/A	Ketchup		Yes
158 F	AB-1 +	No	N/A	N/A	Semen		Yes
160 F	AY-7 −	No	N/A	N/A	Saliva		Yes
162 F	AB-1 +	Yes	Human	N/A	Human		Yes
165 F	AY-7 +	No	NO	N/A	Egg white		Yes
175 F	LCV−	No	N/A	N/A	Egg white		Yes
**4 F**	**None**	**Yes**	**Animal**	**Chicken**	**Chicken**		**Yes**
**24 S**	**None**	**No**	**No**	**N/A**	**Egg**		**Yes**
**25 S**	**LCV+ **	**Yes**	**ANIMAL**	**BOVINE**	**Bovine**		**Yes**
**41 S**	**AB-1 + **	**No**	**N/A**	**N/A**	**Ketchup**		**Yes**
**44 S**	**None**	**Yes**	**ANIMAL**	**PORCINE**	**Wild boar**		**Yes**
**53 S**	**None**	**Yes**	**HUMAN**	**N/A**	**Human blood + EDTA**		**Yes**
**72 S**	**None**	**No**	**N/A**	**N/A**	**Steak sauce**		**Yes**
**76 S**	**AY-7 + **	**No**	**N/A**	**N/A**	**Semen**		**Yes**
**107 F**	**None**	**Yes**	**Animal**	**Porcine**	**Porcine**		**Yes**
**144 F**	**None**	**No**	**N/A**	**N/A**	**Blank**		**Yes**
170 F**	**None**	**No**	**N/A**	**N/A**	**Human blood**		**No**

The Table reports a total of 56 samples, 45 of which were reported in Tables 1 and 2. Originals of samples 13 and 14 were no longer available and could not be re-processed.

*S* stain, *F* fingermarks.

Rows in bold indicate additional new samples analysed after strategy refinement.

*Probable sample mislabelling.

**Blood in trace amounts for which instrumental sensitivity might have been insufficient. BET indicates “blood enhancement technique” and the corresponding column shows “none” for none applied or reports the name of the technique with the enhancement result; “−” indicates no enhancement whereas “+” indicates enhancement. AB-1 (Acid Black 1); LCV (Leucocrystal Violet); AY-7 (Acid Yellow 7).

### Analysis of the final validation set of blind samples

Confirmed markers for human blood, animal blood and semen were taken forward in a “final validation” of the method applied to a representative set of additional 13 enhanced and non-enhanced blind samples including a range of stains and marks in blood, semen, other biofluids and non-biofluid related matrices.

Out of these 13 samples, 12 were correctly identified (Table 6). Once again, there was an instance where a presumptive test incorrectly indicated blood when the sample was instead correctly identified by MALDI MSP as semen (sample 176 F).

**Table 6:** Final validation set of blind samples carried out post strategy change for species determination, showing identity claim and true identity of each sample: 92% correct identification rate Sample in bold indicates a sample correctly indicated as animal blood though animal species was inconclusive.

Sample no.	Blood presumptive test	Presumptive test result	Claim	True identity
15 S	None	NA	Human blood	Human blood
22 S	AY-7	+	Human blood	Human blood
104 F	None	NA	Chicken blood	Chicken blood
121 F	None	NA	Not blood	Saliva
127 F	None	NA	Semen	Semen
128 F	None	NA	Bovine blood	Bovine blood
129 F	AY-7	+	Chicken Blood	Chicken blood
**138 F**	**AY-7**	** + **	**Animal blood − species inconclusive**	**Bovine blood**
146 F	None	NA	Not blood	Beet juice
147 F	None	NA	Semen	Semen
155 F	AY-7	+	Porcine blood	Porcine blood
156 F	None	NA	Porcine blood	Porcine blood
176 F	AY-7	+	Not blood	Semen

*S* stain, *F* fingermark.

The symbol +  (in the presumptive test result column) indicates that the sample tested positive for the presence of blood.

Sample 138 F was correctly classified as animal blood; the myoglobin peptide marker at nominal *m/z* 1593 was detected in this sample but the marker at nominal *m/z* 1764 was not. As a result, the claim of bovine blood was not made. From this, it can be surmised that, in bovine blood, the GAPDH marker at nominal *m/z* 1764 does not need to be present in addition to the myoglobin signal at *m/z* 1593, in order to correctly identify this animal species as the source of blood.

From a forensic perspective, it is important to highlight that these samples were, at the point of the proteolytic digestion and analysis, 3 years old. Therefore the successful application of the strategy devised to these samples opens up the avenue for investigation of cold cases.

## Conclusions

This study aimed at providing validation data to support the previously reported application of MALDI MS and proteomics-based methods for the detection and provenance of blood. The rationale behind the development, improvement and validation of such methods are the reduced analysis time, the easier data acquisition and the user friendly data interpretation. A range of validation samples were prepared blind to the analyst team (including the design of sample collection). Samples included fingermarks and stains prepared with a biofluid (human semen, saliva or sweat), human blood, animal blood (domestic pig, wild boar, chicken and bovine) or biofluid—unrelated matrices. A sample subset was pre-enhanced with either Acid Black 1, Acid Yellow 7 or Leucocrystal Violet. The work presented in this study focuses on an adapted protocol for sample extraction and in solution digestion. However, the approach can easily be transferred to in situ proteomics for both profiling (for pure detection of blood and its provenance) and imaging purposes (when the information of blood presence and provenance is visualised directly on the ridge pattern), as our previous studies have shown.

The present study demonstrates that the overall analytical approach developed enables, regardless of the prior blood enhancement technique used (within the system under investigation): (a) determination or exclusion of blood presence; (b) discrimination between human blood and animal blood; (c) determination of animal blood at a species level; (d) determination of presence (or co-presence) of semen. Notably, in some cases the method was able to correctly refute the positive indication on the presence of blood given by the presumptive test.

Given that semen markers were also found, the MALDI based method is on track to become a multiplexed approach for the screening of biofluids.

A key finding was made with respect to the determination of blood provenance at animal species level. Contrary to our initial hypothesis, that the most abundant and blood specific proteins could be used as molecular targets to prove/disprove the presence of blood and indicate provenance, the relevant markers may instead change according to how the blood was “generated” and “collected”. For example, it was rather surprising that haemoglobin signals in chicken (blind) samples are not detected and only one is detected (poorly) for bovine and porcine blood; conversely, GAPDH a non-blood specific protein is detected in the animal blind samples but not within the blood deriving from intravenous collection.

Indeed, the animal mass spectral profiles of the blind samples (collected by a butcher from the blood filling the heart cavity of the animal) greatly differed from (i) those obtained for the corresponding intravenous blood, (ii) those of the blood residues from packaged meat and (iii) those of the blood originating from butcher prepared meat. It was also evident that for the last two types of “blood systems”, a greater instrumental sensitivity is desirable due to the extreme blood dilution and the use of preservatives (for packaged meat). For animal species determination, the sample preparation design for the next piece of research should consider the most common scenarios in which animal blood may be found. Perhaps the most commonly encountered scenario is blood contamination from handling packaged and butcher prepared meat and, in a less common scenario, use of tools for hunting and dressing game. However, animals being shot or stabbed at a crime scene would produce a “blood system” resembling more the composition of intravenous blood. All of these “blood systems” require a thorough investigation and multivariate statistical analysis could provide more rapid answers as to the provenance markers which can then be subsequently identified. In a subsequent full validation study will use the knowledge generated by the pre-validation study presented here, in a much larger project. This will include a higher number of surfaces of deposition, donors, blood enhancement techniques and the possibility to lift blood fingermarks for imaging purposes when the surface of deposition is not directly amenable to MALDI MS Imaging. An exploration of blood composition and biomarkers from additional animal species will provide further versatility to the applicability of the method.

## Supplementary information

Supplementary Information.

## Data Availability

The datasets generated during and/or analysed during the current study are available from the corresponding author on reasonable request.
