# Supplementary material for: Pre-validation of a MALDI MS proteomics-based method for the reliable detection of blood and blood provenance
Source: Sci Rep. 2020 Oct 13;10:17087. doi: 10.1038/s41598-020-74253-z (PMC7555906; doi:10.1038/s41598-020-74253-z)
Supplement: Supplementary file 1 — Supplementary Information. [file 41598_2020_74253_MOESM1_ESM.pdf]

Pre-validation of a MALDI MS proteomics-based method for the reliable detection of blood and blood provenance

Katherine Kennedy[1#], Cameron Heaton[1#], Glenn Langenburg[2], Laura Cole[1], Tom Clark[3], Malcolm R. Clench[1], Vaughn Sears[4], Mark Sealey[5], Richard McColm[5] and Simona Francese[1*]

[1]Centre for Mass Spectrometry Imaging, Biomolecular Research Centre, Sheffield Hallam University, Sheffield, UK
[2] Elite Forensic Services, LLC, Saint Paul, Minnesota, USA
[3] Sheffield Hallam University, Sheffield, UK
[4]Formerly from CAST, Home Office, St Albans, UK
[5] Defence Science and Technology Laboratories (DSTL), Porton Down, Salisbury, UK

[#] These authors have equally contributed to the paper.
* Corresponding author: s.francese@shu.ac.uk

Tables

Intravenous BOVINE blood -peptide *m/z*	Mass accuracy (ppm)	Blind sample 38 (BOVINE) - peptide *m/z*	Mass accuracy (ppm)
**αHB** 1071.554	0.6	---	
**αHB** 1529.734	0.4	---	
**αHB** 1833.892	-0.4	---	
**βHB** 1265.828	-2.2	---	
**βHB** 1274.723	-1.9	**βHB** 1274.706	15
**βHB** 1422.716	-7.6	---	
**βHB** 1477.801	-0.7	---	
**βHB** 1752.898	-0.6	---	
**βHB** 1868.951	-1.6	---	
		**GAPDH** 795.418	-3.0
		**GAPDH** 805.432	1.1
		**GAPDH** 1032.595	-6.6
		**GAPDH** 1358.681	-9.5
		**GAPDH** 1369.743	-5.5
		**GAPDH** 1369.743	-5.5
**GAPDH** 1499.776	-8.2		
		**GAPDH** 1499.789	-7.2
		**GAPDH** 1615.880	2.5
		**GAPDH** 1763.802	2.5
		**myoglobin** 629.343	3.9
		**myoglobin** 748.433	-3.2
		**myoglobin** 1271.668	3.8
		**myoglobin** 1393.821	3.8
		**myoglobin** 1592.838	-0.7

		**myoglobin** 1669.834	-1.3
**Intravenous PORCINE blood -peptide *m/z***	**Mass accuracy (ppm)**	**Blind sample 7 (PORCINE) - peptide *m/z***	**Mass accuracy (ppm)**
**αHB 1041.540**	-3.6	---	
**αHB 1115.631**	-10.1	---	
**αHB** 1422.704	-3.2	---	
**αHB** 1628.904	-4.8	---	
**αHB** 1876.893	-2.3	---	
**αHB** 1935.972	-3.2	---	
**βHB** 1265.830	-2.2	---	
**βHB** 1274.726	-1.9	---	
**βHB** 1449.796	-7.6	---	
**βHB** 1866.012	-7.2	---	
		**GAPDH** 795.422	4.3
		**GAPDH** 977.547	5.4
**GAPDH** 1499.778	7.4		
		**GAPDH** 1763.813	6.1
		**myoglobin** 1592.820	-11.9

Table S1. Putative identification of haemoglobin, glyceraldehyde 3-phosphate hydrogenase (GAPDH) and myoglobin in blind porcine and bovine samples versus the corresponding intravenous blood samples.

Figures

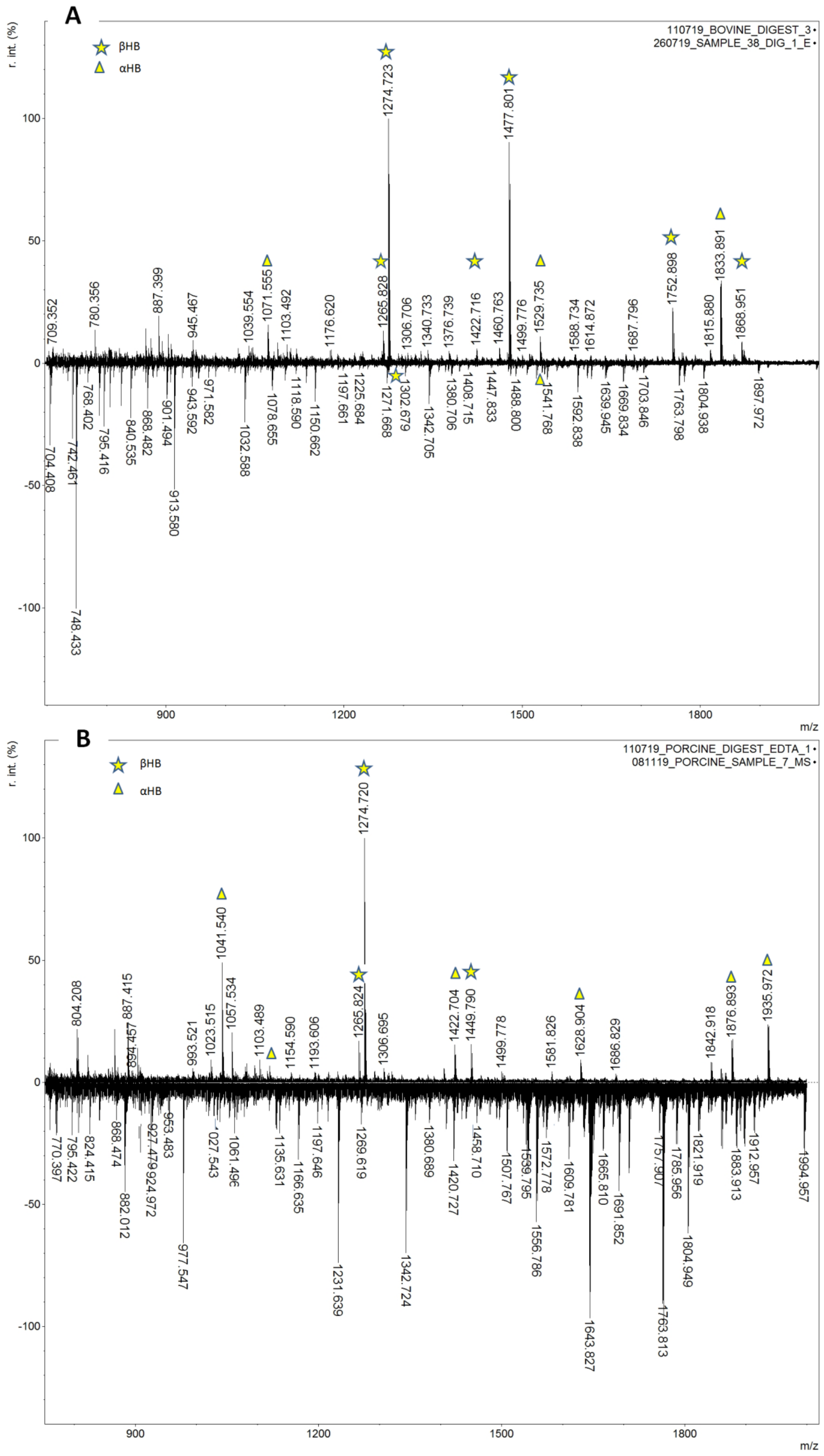
A
B
r. int. (%)
βHB
αHB
110719_BOVINE_DIGEST_3
260719_SAMPLE_38_DIG_1_E
110719_PORCINE_DIGEST_EDTA_1
081119_PORCINE_SAMPLE_7_MS
100
50
0
-50
-100
900
1200
1500
1800
m/z
709.352
780.356
887.399
945.467
1039.554
1071.555
1103.492
1176.620
1265.828
1274.723
1306.706
1340.733
1376.739
1422.716
1460.763
1477.801
1499.776
1529.735
1588.734
1614.872
1687.796
1752.898
1815.880
1833.891
1868.951
704.408
742.461
748.433
768.402
795.416
840.535
868.482
901.494
913.580
943.592
971.582
1032.588
1078.655
1118.590
1150.662
1197.661
1225.684
1271.668
1302.679
1342.705
1380.706
1408.715
1447.833
1488.800
1541.768
1592.838
1639.945
1669.834
1703.846
1763.798
1804.938
1897.972
804.208
864.457
887.415
993.521
1023.515
1041.540
1057.534
1103.489
1154.590
1193.606
1265.824
1274.720
1306.695
1422.704
1449.790
1499.778
1581.826
1628.904
1686.829
1842.918
1876.893
1935.972
770.397
795.422
824.415
868.474
882.012
924.972
953.483
977.547
1061.496
1135.631
1166.635
1197.646
1231.639
1269.619
1342.724
1380.669
1420.727
1458.710
1507.767
1556.786
1559.795
1572.778
1609.781
1643.827
1665.810
1691.852
1757.907
1763.813
1785.956
1804.949
1821.919
1883.913
1912.957
1994.957

Fig. S1. MALDI MS spectral comparison of intravenous reference blood and blind blood samples for bovine (A) and porcine (B). The spectral profiles appear different in both cases with haemoglobin (HB) signals only present in the intravenous samples but not in the blind samples.

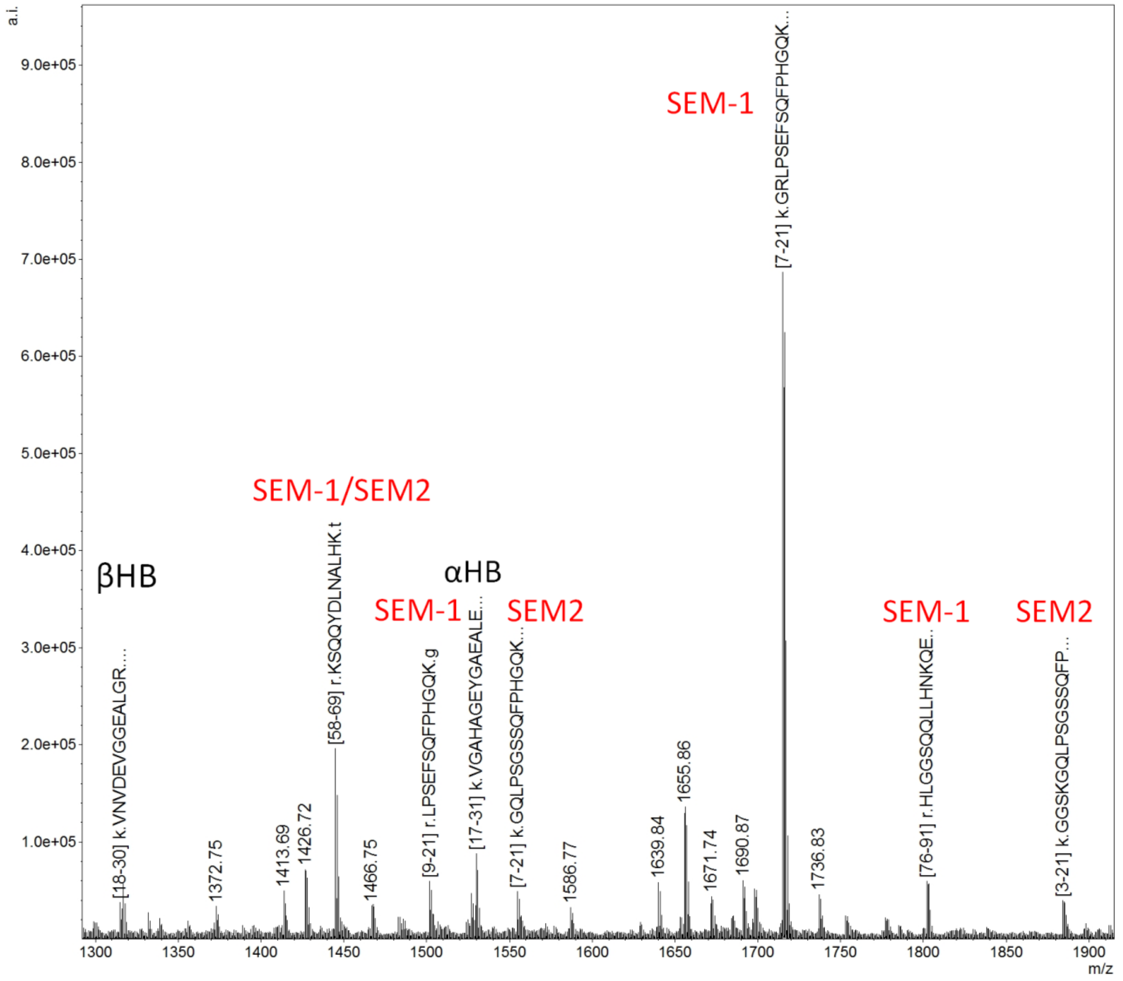

Fig. S2. MALDI MS spectrum of blind sample 32 (Semen). Haemoglobin and semenogelin 1 and 2 ion signals were putatively assigned except for *m/z* 1714.85 which was confirmed by MS/MS.

Accession numbers list of putatively identified and confirmed proteins

Bovine GAPDH (UniProtKB-P10096)

Bovine Haemoglobin (alpha chain) (UniProtKB - P01966)

Bovine Haemoglobin (beta chain) (UniProtKB - P02070)

Bovine Myoglobin (UniProtKB - P02192)

Chicken GAPDH (UniProtKB- P00356)

Chicken Haemoglobin (alpha chain) (UniProtKB - P01994)

Chicken Haemoglobin (beta chain) (UniProtKB - P02112)

Human Erythrocyte protein band 4.2 (UniProtKB - P16452)

Human Semenogelin -1(UniProtKB - P04279)

Human Semenogelin-2 (UniProtKB - P04279)

Human Haemoglobin (alpha chain) (UniProtKB - P69905)

Human Haemoglobin (beta chain) (UniProtKB - P68871)

Porcine GAPDH (UniProtKB - P00355)

Porcine Haemoglobin (alpha chain) (UniProtKB - P01965)

Porcine Haemoglobin beta chain) (UniProtKB - P02067)

Porcine Myoglobin (UniProtKB - P02189)
